# Burnout and fatigue amongst internal medicine residents: A cross-sectional study on the impact of alternative scheduling models on resident wellness

**DOI:** 10.1371/journal.pone.0291457

**Published:** 2023-09-14

**Authors:** Jack H. Yuan, Yiming Huang, Brianna K. Rosgen, Sarah Donnelly, Xiaoyang Lan, Steven J. Katz

**Affiliations:** 1 Department of Medicine, University of Alberta, Edmonton, Alberta, Canada; 2 Cumming School of Medicine, University of Calgary, Calgary, Alberta, Canada; Ospedale Pediatrico Bambino Gesu, ITALY

## Abstract

**Background:**

Fatigue and burnout are prevalent among resident physicians across Canada. Shifts exceeding 24 hours are commonly purported as detrimental to resident health and performance. Residency training programs have employed strategies towards understanding and intervening upon the complex issue of resident fatigue, where alternative resident scheduling models have been an area of active investigation. This study sought to characterize drivers and outcomes of fatigue and burnout amongst internal medicine residents across different scheduling models.

**Methods:**

We conducted cross-sectional surveys were among internal medicine resident physicians at the University of Alberta. We collected anonymized socioeconomic demographics and medical education background, and estimated associations between demographic or work characteristics and fatigue and burnout outcomes.

**Results:**

Sixty-nine participants competed burnout questionnaires, and 165 fatigue questionnaires were completed (response rate of 48%). The overall prevalence of burnout was 58%. Lower burnout prevalence was noted among respondents with dependent(s) (p = 0.048), who identified as a racial minority (p = 0.018), or completed their medical degree internationally (p = 0.006). The 1-in-4 model was associated with the highest levels of fatigue, reported increased risk towards personal health (OR 4.98, 95%CI 1.77–13.99) and occupational or household harm (OR 5.69, 95%CI 1.87–17.3). Alternative scheduling models were not associated with these hazards.

**Conclusions:**

The 1-in-4 scheduling model was associated with high rates of resident physician fatigue, and alternative scheduling models were associated with less fatigue. Protective factors against fatigue are best characterized as strong social supports outside the workplace. Further studies are needed to characterize the impacts of alternative scheduling models on resident education and patient safety.

## Introduction

Fatigue and burnout are concerns consistently reported by resident physicians across North America [1–3]. With increasing recognition towards the impact of these concerns as they relate to mental health, patient safety, and resident education, residency programs across the country have been prompted to re-evaluate how resident training is executed. Of particular interest in recent literature has been the longstanding use of in-hospital shifts exceeding 24 hours, which have been posed as an occupational hazard towards resident physician health and safety [4–7]. Notably, 24 hours of sustained wakefulness results in cognitive impairment equivalent to blood alcohol levels over the legal driving limit [8]. Various studies also demonstrate worsening sentiments of isolation and empathy among residents who work with these sleep disturbances [6–8]. As such, safe delivery of patient care under these circumstances has been put to question and continues to be a growing area of study [9–13]. Notably, a recent longitudinal study that followed over 4800 senior resident physicians over 8 years has shown a dose-dependent increase in risk of self-reported medical error, preventable adverse events, occupational and household injury, and attentional failures when residents worked greater than 48 hours per week, with risk doubling at over 60 hours per week [14].

While the utilization of 24-hour shifts is steeped in tradition, evidence such as this is increasingly alerting medical leadership to the need for change. In Canada, the Royal College of Physicians and Surgeons of Canada (RCPSC) published a national consensus on resident duty hours in 2013 [9]. Although recommending that shifts greater than 24 hours be avoided, they concluded that existing evidence, at the time, was not sufficient to support a national limit on duty hours as the impacts on resident training and education are unclear. Nonetheless, they advocated for innovation in alternative models for resident call scheduling [9]. Furthermore, the RCPSC also recognized that resident wellness and fatigue are complex multidimensional issues and highlighted the importance of strategies to manage fatigue on a global scale [9].

More literature characterizing the causes and outcomes of resident fatigue is needed, in particular as these issues relate to scheduling and duty hours. Alternative scheduling models have been implemented across different medical and surgical specialties previously and often include various shift-based strategies. One particular example is night float, a model that has increasingly become the subject of study with respect to resident well-being, educational outcomes, and patient safety [15–17]. We sought to further examine the drivers and impacts of both fatigue and burnout amongst the internal medicine residency program in Edmonton, Alberta, Canada, in the context of various scheduling models including night float.

## Methods

We conducted cross-sectional surveys among internal medicine residents at the University of Alberta in post-graduate years (PGY) one through three. 115 active resident physicians were invited to complete three monthly surveys from September to November, 2022. Participants were rotating monthly through various clinical settings within internal medicine and its specialties, with five different scheduling models:

1. 1-in-4 call. Residents complete in-hospital 24-hour shifts on average every four days during the rotation, typically a maximum of seven shifts per month. Two additional hours on service are allotted for patient handover. Residents return to service following a day off post-call. Duty hours approximate to 65–70 hours per week.
2. Late-stay call. Residents extend one typical workday each week to 2100 hrs (13-hour daytime shift), and complete two in-hospital 24-hour shifts and one 13-hour day shift over two weekends in the month. Duty hours approximate to 55–60 hours per week.
3. Home call. In addition to regular hours, residents cover one to two overnight shifts from home per week, as well as one weekend consisting of daytime rounds and overnight home call. Services with this model was limited to primarily ambulatory subspecialties such as rheumatology and infectious diseases. Duty hours approximate to 40–45 hours per week in-hospital.
4. Night float. Over a two-week period, residents provide weekday overnight in-hospital coverage for internal medicine or a subspecialty, with shifts either 8 or 12 hours. Duty hours approximate to 45–60 hours per week.
5. No call. Residents have no call requirements, but may be called upon to fill gaps in call coverage, to a maximum of one or two shifts per month. Duty hours approximate to 35–40 hours per week.

### Survey

Surveys were administered using the Google Forms platform. Informed consent was obtained before each survey, and responses were anonymous and voluntary. Each administration was implemented using a modified Dilman’s approach, with invitations one week prior to survey opening, followed by two reminders prior to the completion deadline. Participants were provided a gift card monetary incentive. STrengthening the Reporting of OBservational studies in Epidemiology (STROBE) reporting guidelines were followed [18]. The University of Alberta Health Research Ethics Board provided ethical approval, Pro00119275.

### Demographics

Information on age, gender (i.e., female, male, non-binary), post-graduate year, marital status (i.e., single, in a relationship, cohabiting, married, separated), medical training (i.e., Canadian medical graduate [CMG], and international medical graduate [IMG]), and whether participants had dependents, or identified as a racial minority were collected. Specific rotation completed and associated scheduling models were collected.

### Burnout

Burnout metrics were collected using the Maslach Burnout Inventory—Human Services Survey for Medical Personnel (MBI-HSS) in the first survey administration only. The instrument contains 22 items scored from 0 to 6 based on self-reported frequency of agreement, and divides burnout into three domains: emotional exhaustion, depersonalization, and low personal accomplishment [19]. Burnout was defined as emotional exhaustion scores ≥27, or depersonalization scores ≥10, consistent with criteria used in existing literature [20]. Low personal accomplishment (≥33) scores were evaluated independently of burnout.

### Fatigue metrics

Subjective measures of levels and impacts of fatigue were collected. The Swedish Occupational Fatigue Inventory (SOFI) was selected as an instrument to assess fatigue, given prior use to study healthcare professionals [21]. The instrument assesses five domains: lack of energy, lack of motivation, sleepiness, physical exertion, and physical discomfort. The SOFI was modified to a single self-report of participants’ experience of each domain for simplicity. Average number of hours of sleep attained on-call and at home, if applicable, were collected. Subjective drivers of fatigue were collected and assigned up to three themes, with common themes grouped into major identified drivers of fatigue. The impact of fatigue was assessed with Likert-style questions involving self-reported risk of medical error, unprofessionalism, occupational or household harm, negative impact on relationships, and disruption to maintenance of personal health.

### Statistical analysis

STATA 14.2 (StataCorp, TX) was used for all statistical analyses. Demographic characteristics were summarized using frequencies, descriptive statistics, and accompanying variability estimates. Burnout proportions was compared using t-tests and chi-squared tests for binary and categorical variables, respectively. Multivariable logistic (burnout) and ordinal (fatigue) regression models were used to estimate associations between explanatory variables and outcomes. Explanatory variables were selected based on theoretical significance and significance in previous literature, including: age, gender, post-graduate year, marital status, dependents, racial minority status, medical training, and scheduling model [22–24]. In all analyses, the no call model was used as the base comparison between scheduling models. The rationale for this was that understanding resident experiences of scheduling models require consideration of chronicity with each model. There would be difficulty comparing 1-in-4 call directly to night float given that a resident may only be required to complete two to four weeks of night float in place of multiple months of the 1-in-4 scheduling model. The objective was thus to characterize each scheduling model’s impact on resident wellness compared to baseline resident health. The correlation between burnout and fatigue was assessed using Kendall’s Tau. Listwise deletion was used to handle missing values. A two-sided alpha level of 0.05 was used to determine statistical significance.

## Results

The response rate was 48% (165/345 available responses from 115 residents). Demographic characteristics, scheduling models, and associated burnout prevalence’s of the study population are shown in Table 1. The median age was 28 years old (IQR 27–30).

**Table 1:** Demographic and work characteristics of respondents.

Variable		Total Responses N (%)	Burnout prevalence N (%)
**Age** ^a^	25–29	110 (71.9%)	30 (66.7%)
	30–34	35 (22.9%)	7 (41.2%)
	35–39	5 (3.2%)	0 (0%)
	40+	3 (2.0%)	0 (0%)
**Gender**	Female	87 (52.7%)	21 (61.8%)
	Male	76 (46.1%)	19 (55.9%)
	Non-binary	0 (0.0%)	0 (0.0%)
	Prefer not to answer	2 (1.2%)	0 (0.0%)
**Post-Graduate Year** ^b^	PGY-1	51 (31.2%)	10 (58.8%)
	PGY-2	56 (34.4%)	15 (57.7%)
	PGY-3	56 (34.4%)	14 (58.3%)
**Marital Status** ^b^	Single	36 (22.1%)	10 (52.6%)
	Non-cohabiting relationship	35 (21.5%)	9 (64.3%)
	Cohabiting relationship	47 (28.8%)	13 (76.5%)
	Married	45 (27.6%)	7 (38.9%)
**Dependents** ^c^	No dependents	130 (79.3%)	36 (64.3%)
	Dependents	34 (20.7%)	4 (33.3%)
**Racial Minority** ^c^	Yes	95 (58.3%)	19 (76.0%)
	No	68 (41.7%)	20 (46.5%)
**Status before entering program** ^c^	CMG	129 (78.7%)	36 (65.5%)
	IMG	35 (21.3%)	3 (23.1%)
**Scheduling model**	No call	51 (30.9%)	13 (54.2%)
	Home call	20 (12.1%)	5 (55.6%)
	Night float	19 (11.6%)	4 (57.1%)
	Late stay/weekend call	37 (22.4%)	8 (53.3%)
	Traditional 1-in-4	38 (23.0%)	10 (71.4%)

^a^ n = 12 missing;

^b^ n = 2 missing;

^c^ n = 1 missing;

^d^ p-values comparing proportion of burnout between categories.

### Burnout and personal accomplishment

Sixty-nine distinct participants responded to the MBI-HSS, and the overall prevalence of burnout was 57.9% (95%CI 45.8–69.2%). In univariate analyses, respondents who had dependent(s) (33.3% vs. 64.3% p = 0.048), who identified as a racial minority (46.5% vs. 76.0%, p = 0.018), and IMG respondents (23.1% vs. 65.5%, p = 0.006) had a significantly lower prevalence of burnout. Differences in burnout among differing marital status (p = 0.139), age (p = 0.106), gender (p = 0.440), and years of residency (p = 0.997) were not statistically significant. Across scheduling models, 1-in-4 call had the highest prevalence of burnout (71.4%), followed by night float (57.1%), home call (55.6%), no call (54.2%), and late stay call (53.3%), though these differences did not reach statistical significance.

**Fig 1:**
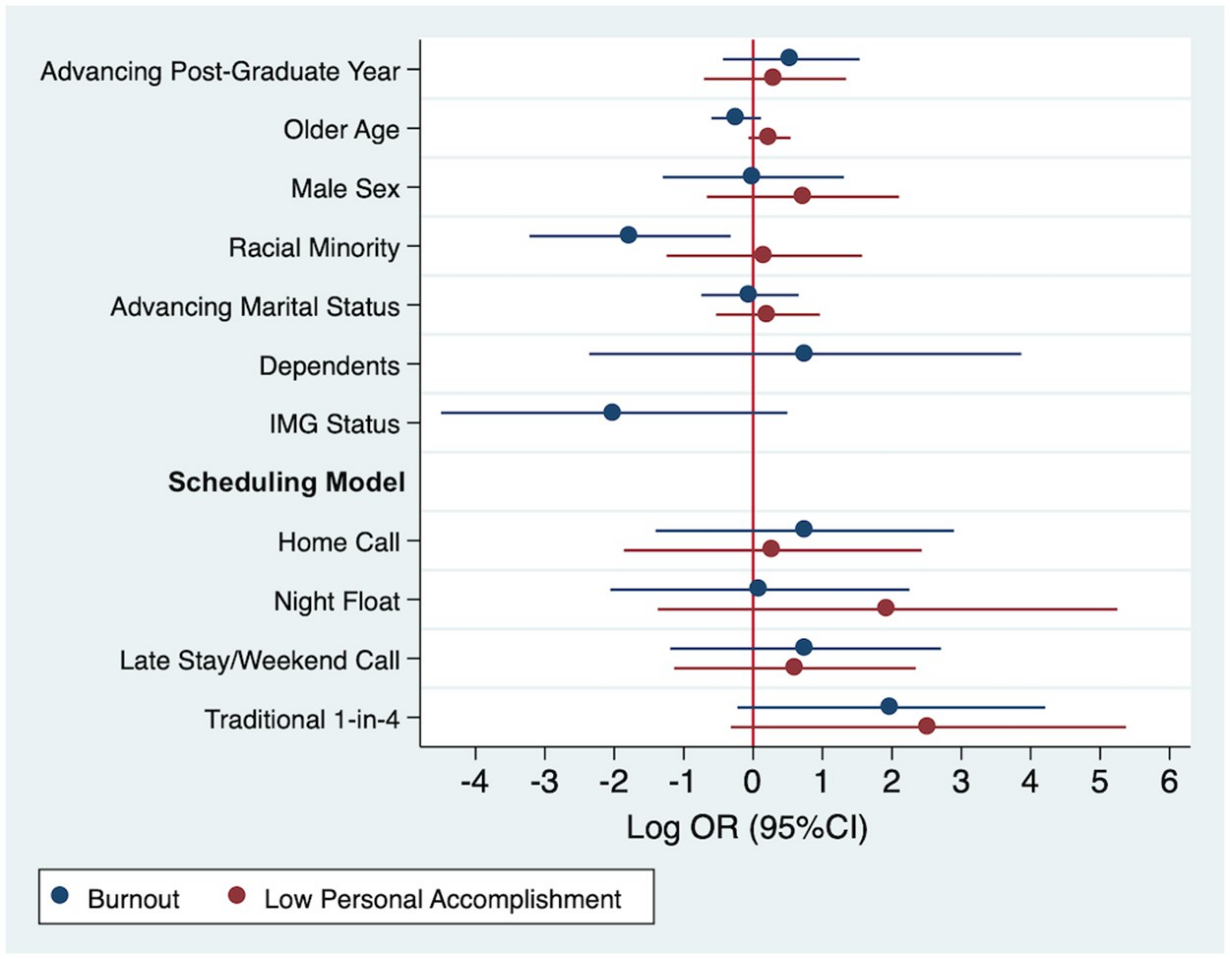
Plot of multivariable associations (Log Odds Ratio, 95% Confidence Interval) between demographic/work characteristics and burnout and low-personal accomplishment outcomes. Low personal accomplishment ORs for having dependents and IMG status were not plotted due to extreme values (Dependents OR 1.04e^6^ 95%CI 0 − ∞; IMG Status OR 9.98e^-9^, 95%CI 0 − ∞).

Multivariable odds ratios for burnout and personal accomplishment were calculated for demographic and work variables using logistic regression models and shown in Fig 1. Being a racial minority was significantly associated with decreased burnout (OR 0.17, 95%CI 0.04–0.72). All other associations with burnout were not statistically significant. Low personal accomplishment was not significantly associated with any demographic or work factors, however the odds ratios were highest among 1-in-4 call (OR 12.50, 95%CI 0.73–215.37) and night float (OR 6.94, 95%CI 0.25–190.42).

### Fatigue metrics

**Fig 2:**
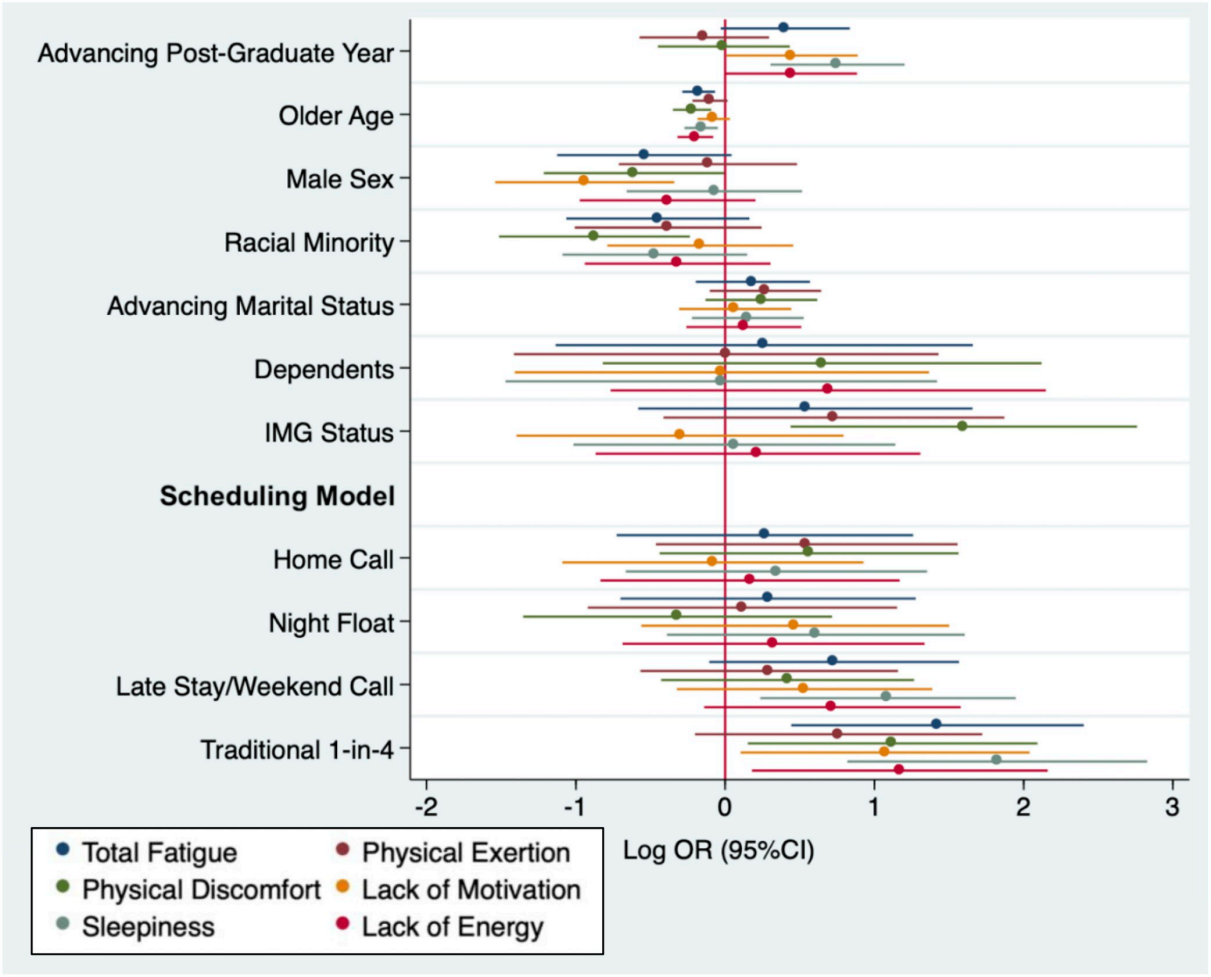
Plot of multivariable associations (Log Odds Ratio, 95% Confidence Interval) between demographic/work characteristics and total fatigue and SOFI fatigue domains.

Higher burnout scores were strongly correlated with higher fatigue scores (Kendall’s Tau = 0.40). Odds ratios for high fatigue scores, as measured by the SOFI, with respect to demographic characteristics and scheduling models are shown in Fig 2.

1-in-4 call was associated with higher levels of both total fatigue (OR 4.15, 95%CI 1.56–11.05) and four individual SOFI domains, physical discomfort (OR 3.07, 95%CI 1.16–8.10), lack of motivation (OR 2.92, 95%CI 1.11–7.69), sleepiness (OR 6.19, 95%CI 2.27–16.90), lack of energy (OR 3.22, 95%CI 1.20–8.68). Older age was associated with less total fatigue (OR 0.84, 95%CI 0.75–0.93), physical discomfort (OR 0.80, 95%CI 0.71–0.91), sleepiness (OR 0.85, 95%CI 0.76–0.95), and lack of energy (OR 0.82, 95%CI 0.73–0.92). Male sex was associated with less lack of motivation (OR 0.39, 95%CI 0.21–0.71). Being a racial minority was associated with less physical discomfort (OR 0.41, 95%CI 0.22–0.79), while IMG status was associated with higher levels of physical discomfort (OR 4.95, 95%CI 1.55–15.80). Late stay/weekend call was associated with increased sleepiness (OR 2.98, 95%CI 1.27–7.00).

The mean (±SD) hours of cumulative sleep obtained per 24-hour call shift reported was 1.6 (± 1.5) hours. Hours slept while not on call was not significantly different across scheduling models (mean hours slept = 6.8 ± 1.1 hours, p = 0.638).

### Drivers of fatigue

**Fig 3:**
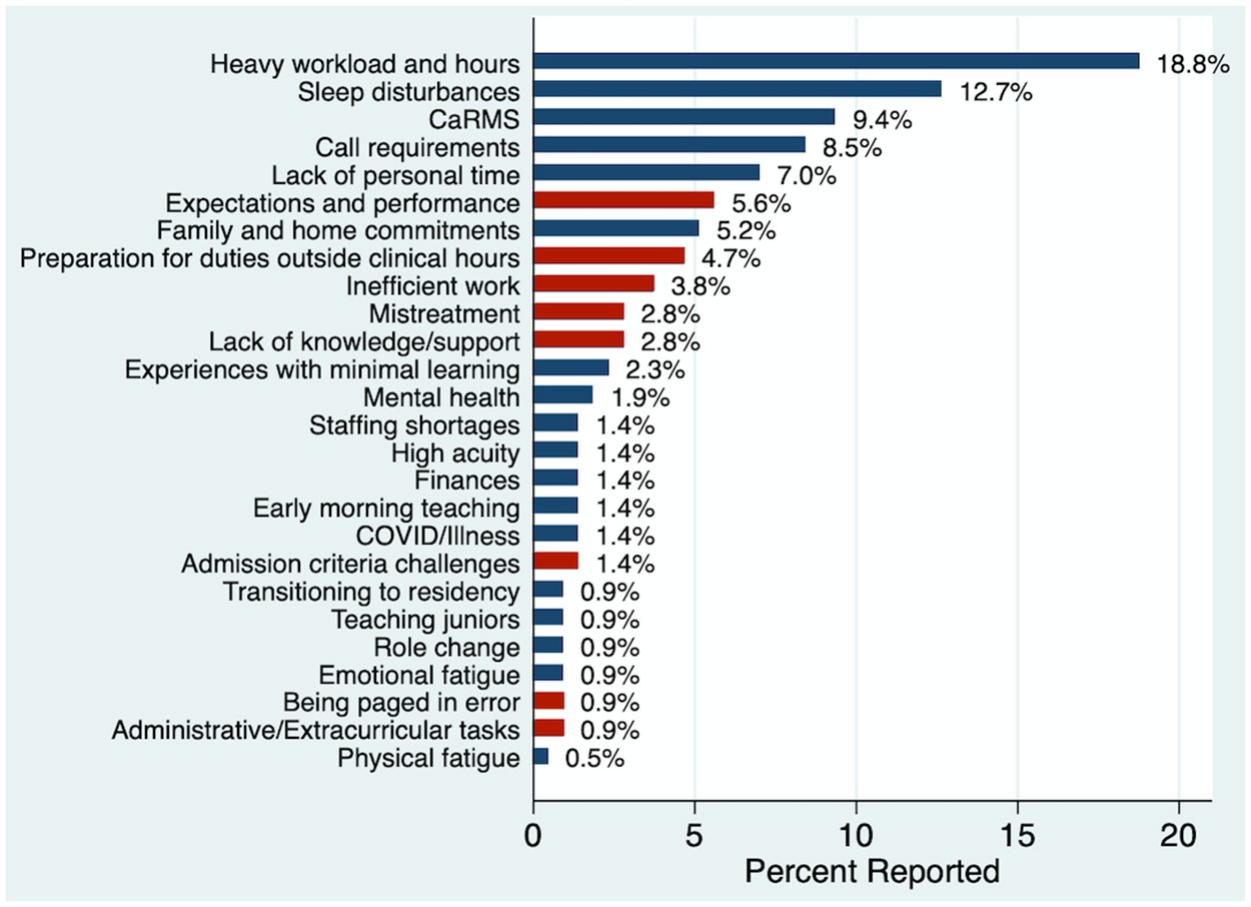
Percentage (%) of reported subjective drivers of fatigue across n = 148 responses. Red bars indicate identified readily reversible drivers of fatigue. “CaRMS” denotes the Canadian Residency Matching Service, which resident physicians in Canadian programs apply for their subspecialty match in their PGY-3 year.

The most common reported drivers of fatigue are shown in Fig 3, with reversible drivers of fatigue indicated. Twenty-five themes were identified from the responses.

### Impacts of fatigue

**Fig 4:**
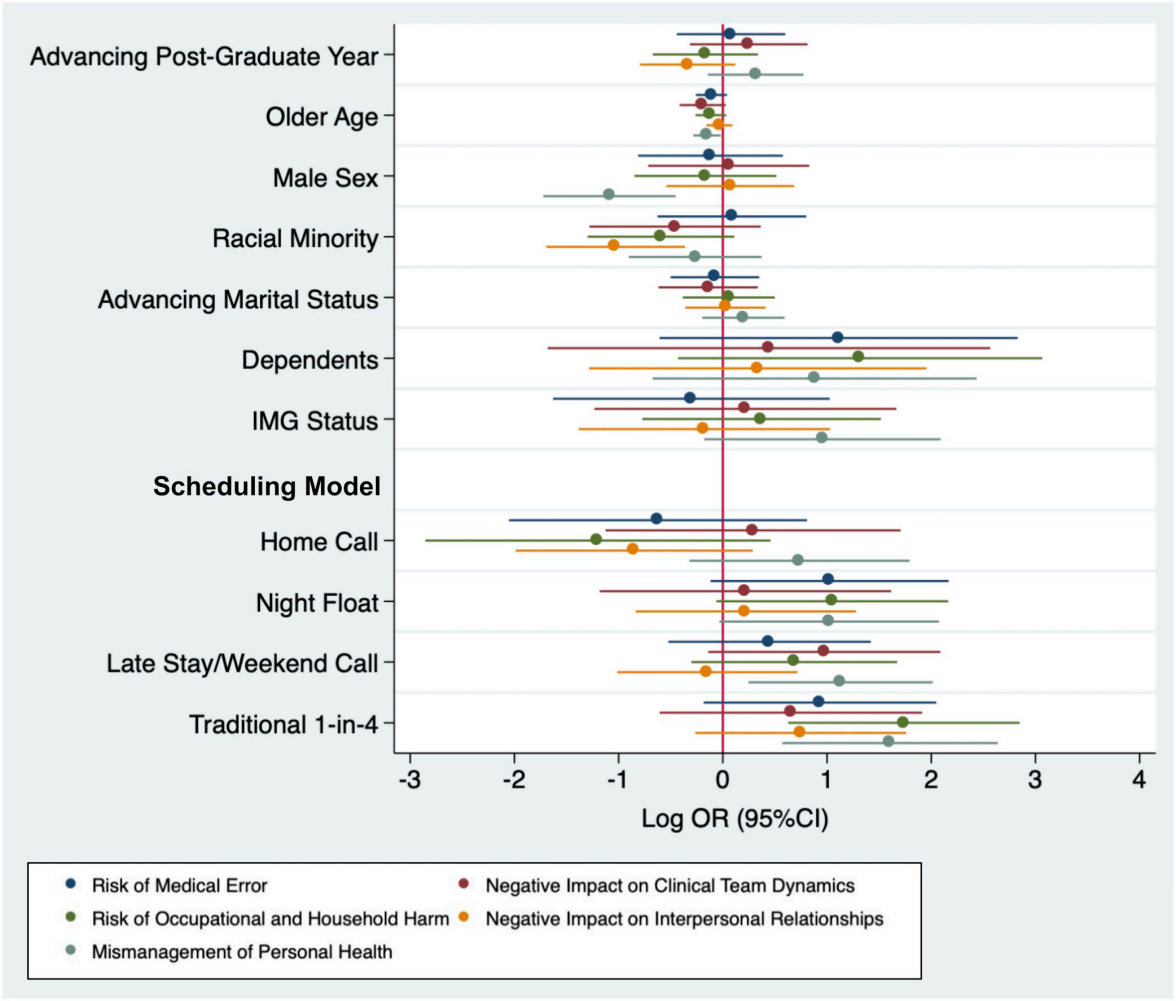
Plot of multivariable associations (Log Odds Ratio, 95% Confidence Interval) between demographic/work characteristics and impacts of fatigue.

Aggregate responses to Likert-style questions regarding the consequences of fatigue are shown in Fig 4. 1-in-4 call was associated with an increased perceived risk of occupational and household harm (OR 5.69, 95%CI 1.87–17.3) and mismanagement of personal health (OR 4.98, 95%CI 1.77–13.99). Late stay/weekend call was associated with increased perceived mismanagement of personal health (OR 3.09, 95%CI 1.28–7.49). Older age (OR 0.86, 95%CI 0.75–0.98) and male sex (OR 0.34, 95%CI 0.18–0.64) was associated with less perceived mismanagement of personal health. Being a racial minority was associated with less perceived negative impacts on interpersonal relationships (OR 0.36, 95%CI 0.18–0.70).

## Discussion

High rates of both resident and attending physician burnout have been widely reported prior to the COVID-19 pandemic [2, 20, 25], with recent studies demonstrating continually rising rates [23, 26–28]. The prevalence of burnout was 58% in this study, which is consistent with existing literature and reemphasizes the magnitude of the physician burnout epidemic. Fatigue has not been well quantified amongst resident physicians using validated instruments. While aggregate resident perceptions of the impact of fatigue on well-being suggests that absolute fatigue measures are high, our focus lies in identifying individual and work-related factors that may be associated with burnout and fatigue.

Older age and having a spouse or dependents was associated with the lowest fatigue and burnout scores. While some studies have suggested that increasing family commitments (i.e., marriage and children) may be a risk factor [29], our data aligns with literature suggesting that strengthened support system as residents enter these stages of life outweigh increased home commitments [30–32].

There is growing evidence suggesting that duty hours greater than 48 hours per week, burnout and fatigue are associated with increased risk of self-reported medical error, unprofessionalism, and other harms to resident health [14, 33–37]. However, recent work by Taylor *et al*. suggests that resident physicians have yet to conceptualize fatigue as an occupational threat and may still perceive it as a personal challenge [38]. Our study shares similar sentiments, with residents reporting minimal impact on their patient care or professionalism due to burnout or fatigue. Contrary to existing literature, it seems that residents interpret burnout and fatigue to cause more harm to themselves than the patients they care for or the medical institution. With the RCPSC mandating fatigue risk management plans as an accreditation standard for residency programs, fatigue and burnout education will be a key step in contextualizing interventions aimed at reducing resident fatigue [39].

Resident duty hours and their impact on fatigue, patient safety, and educational outcomes have been a contentious topic of global debate in recent years [40–42]. The landscape has been polarizing, on one hand with studies examining patient endpoints such as mortality and length-of-stay with equivocal results, and on another hand a growing body of research expressing resident concerns for patient and their own safety with respect to excessive duty hours [11, 12, 14, 43, 44]. A 2015 systematic review concluded that duty hour restrictions alone have not demonstrated significant benefit to resident burnout or patient safety and may have unintended consequences to educational outcomes [45]. However, excessive duty hours, heavy workloads, and sleep disturbances were the top self-reported drivers of fatigue in our survey, greatly exceeding all other reported factors. It remains clear that duty hours, and scheduling models by extension, remain on the forefront of residents’ thinking in the conversation of burnout and fatigue, requiring further deliberation at the systemic level.

Our results suggest that the 1-in-4 scheduling model was the most harmful to resident physician wellness compared to baseline resident health, with significantly more fatigue across SOFI domains. Importantly, the alternative late-stay and night float models were not associated with these same hazards. Increased integration of alternative scheduling models into residency programs may thus protect against the risks of the 1-in-4 model, and a means to reduce burnout and fatigue in resident physicians. Many previous studies evaluating both well-being and educational outcomes of night float have reported an overall negative impact to resident physicians [16, 17, 46]. Decreased sleep at home and disrupted circadian rhythm were purported to be the primary deleterious impacts on fatigue, and decreased exposure to attending physicians as well as daytime learning events as the deleterious impact on resident education. Our study did not show worsened wellness outcomes with night float, which could attributable to more objective measures of burnout and fatigue, as well as shorter durations of night float rotations. While our study did not evaluate educational outcomes of night float, we suggest that it would be critical for future work to weigh the cumulative impact of night float on duty hours and scheduling. Specifically, how the introduction of two to four weeks of night float per resident per year may eliminate several months of 1-in-4 call, with an overall impact of reducing post-call time, increasing daytime learning and attending physician exposure throughout the remainder of the year. A previous assessment of resident and attending perceptions of the implementation of night float in internal medicine has supported this concept [47].

Our study has several limitations. The primary limitation is our small sample size within a single institution, limiting our power to detect significant associations between explanatory variables and outcomes in our regression models. Although our response rate is similar to other survey-based assessments of resident physician wellness, it may introduce participation bias towards those who seek duty hour reform. This bias could have also been enhanced due to our pooled survey analysis methodology. The generalizability of our conclusions outside of internal medicine specialties may be limited for a few reasons. The nature of overnight coverage requirements for internal medicine may lend well to night float, however may not fit into the structure of other training programs. As well, although 1-in-4 call is a widely adopted scheduling model, practical aspects including patient volume, procedures, and other commitments still vary significantly between programs and specialties. Finally, the rotational differences in type of work required may account for changes in burnout and fatigue rates that were not considered in this study.

Overall, our study highlights high rates of burnout and fatigue in a cohort of Canadian internal medicine residents, with both individual-driven and work-driven risk factors at play. Duty hours remain a primary driver of fatigue and burnout as perceived by residents. Scheduling models had a significant impact on resident wellbeing, and increased adoption of alternative scheduling models may be an effective way to mitigate this risk. More comprehensive investigation of these models is required to understand their cumulative educational impact on residents.

## Supporting information

S1 File Study minimal data set including demographic, burnout, and fatigue responses. (DOCX)
