# Supplementary material for: Burnout and fatigue amongst internal medicine residents: A cross-sectional study on the impact of alternative scheduling models on resident wellness
Source: PLoS One. 2023 Sep 14;18(9):e0291457. doi: 10.1371/journal.pone.0291457 (PMC10501672; doi:10.1371/journal.pone.0291457)
Supplement: S1 File — (DOCX) [file pone.0291457.s001.docx]

**Supplemental Table 1.** Demographic data

| **Post-Graduate Year** | **Age** | **Sex** | **Racial Minority** | **Marital Status** | **Dependents** | **Medical School Status** | **Service Model** |
| --- | --- | --- | --- | --- | --- | --- | --- |
| PGY-2 |  | Female | Yes | Married | Yes | IMG | Home call |
| PGY-2 | 27 | Female | No | In a relationship (not cohabitating with partner) | No | CMG | No call |
| PGY-2 | 26 | Male | No | In a relationship (cohabitating with partner) | No | CMG | No call |
| PGY-3 | 26 | Male | No | In a relationship (cohabitating with partner) | No | CMG | Late-stay / Weekend Call (Pulmonary, Nephrology, GI, Hematology) |
| PGY-1 | 27 | Male | Yes | In a relationship (not cohabitating with partner) | No | CMG | 1-in-4 In-house Call (CTU, CCU, ICU, etc) |
| PGY-1 | 26 | Female | No | Single | No | CMG | Home call |
| PGY-1 | 27 | Male | Yes | In a relationship (not cohabitating with partner) | No | CMG | Late-stay / Weekend Call (Pulmonary, Nephrology, GI, Hematology) |
| PGY-3 | 28 | Male | Yes | In a relationship (cohabitating with partner) | No | CMG | No call |
| PGY-1 | 30 | Female | No | Single | No | CMG | Home call |
| PGY-3 | 29 | Female | No | In a relationship (cohabitating with partner) | No | CMG | No call |
| PGY-3 | 28 | Male | No | In a relationship (cohabitating with partner) | No | CMG | Home call |
| PGY-3 | 32 | Female | Yes | Married | Yes | CMG | No call |
| PGY-1 | 26 | Female | Yes | Single | No | IMG | 1-in-4 In-house Call (CTU, CCU, ICU, etc) |
| PGY-2 | 27 | Male | Yes | In a relationship (cohabitating with partner) | No | CMG | Late-stay / Weekend Call (Pulmonary, Nephrology, GI, Hematology) |
| PGY-3 | 28 | Male | No | In a relationship (cohabitating with partner) | No | CMG | Late-stay / Weekend Call (Pulmonary, Nephrology, GI, Hematology) |
| PGY-2 | 33 | Male | No | Married | Yes | CMG | 1-in-4 In-house Call (CTU, CCU, ICU, etc) |
| PGY-1 | 27 | Male | Yes | In a relationship (cohabitating with partner) | No | CMG | 1-in-4 In-house Call (CTU, CCU, ICU, etc) |
| PGY-3 | 28 | Female | Yes | In a relationship (cohabitating with partner) | No | CMG | No call |
|  |  | Male | Yes | Single | No |  | Night Pool/Night Float |
| PGY-2 | 30 | Male | Yes | Married | Yes | IMG | Night Pool/Night Float |
| PGY-2 | 25 | Female | Yes | In a relationship (cohabitating with partner) | No | CMG | Late-stay / Weekend Call (Pulmonary, Nephrology, GI, Hematology) |
| PGY-3 | 34 | Male | Yes | Married | Yes | IMG | No call |
| PGY-2 | 29 | Male | No | Single | No | IMG | 1-in-4 In-house Call (CTU, CCU, ICU, etc) |
| PGY-2 | 29 | Male | Yes | Single | No | CMG | Night Pool/Night Float |
| PGY-1 |  | Female |  | Married | No | CMG | 1-in-4 In-house Call (CTU, CCU, ICU, etc) |
| PGY-2 | 25 | Female | Yes | In a relationship (not cohabitating with partner) | No | CMG | Late-stay / Weekend Call (Pulmonary, Nephrology, GI, Hematology) |
| PGY-3 | 32 | Female | No | In a relationship (cohabitating with partner) | No | CMG | No call |
| PGY-2 | 28 | Female | No | Single | No | CMG | No call |
| PGY-3 | 30 | Female | Yes | In a relationship (not cohabitating with partner) | No | CMG | Home call |
| PGY-1 | 28 | Female | Yes | Married | Yes | CMG | 1-in-4 In-house Call (CTU, CCU, ICU, etc) |
| PGY-2 | 27 | Female | Yes | Single | No | CMG | No call |
| PGY-2 | 33 | Female | No | Prefer not to answer | No | CMG | No call |
| PGY-2 | 28 | Male | Yes | Single | No | CMG | 1-in-4 In-house Call (CTU, CCU, ICU, etc) |
| PGY-2 | 27 | Female | No | Married | No | CMG | No call |
| PGY-2 | 25 | Male | Yes | In a relationship (not cohabitating with partner) | No | CMG | No call |
| PGY-3 | 28 | Male | No | In a relationship (not cohabitating with partner) | No | CMG | No call |
| PGY-2 | 26 | Male | Yes | Single | No | CMG | 1-in-4 In-house Call (CTU, CCU, ICU, etc) |
| PGY-3 | 27 | Female | Yes | In a relationship (not cohabitating with partner) | No | CMG | Late-stay / Weekend Call (Pulmonary, Nephrology, GI, Hematology) |
| PGY-1 | 27 | Male | Yes | In a relationship (not cohabitating with partner) | No | CMG | 1-in-4 In-house Call (CTU, CCU, ICU, etc) |
| PGY-1 |  | Male | Yes | Married | Yes | IMG | Home call |
| PGY-1 | 34 | Male | No | Married | Yes | IMG | 1-in-4 In-house Call (CTU, CCU, ICU, etc) |
| PGY-1 | 26 | Female | Yes | In a relationship (cohabitating with partner) | No | CMG | Late-stay / Weekend Call (Pulmonary, Nephrology, GI, Hematology) |
| PGY-1 | 27 | Male | Yes | Single | No | CMG | 1-in-4 In-house Call (CTU, CCU, ICU, etc) |
| PGY-2 | 29 | Female | Yes | In a relationship (not cohabitating with partner) | No | CMG | No call |
| PGY-1 | 28 | Male | Yes | Single | No | IMG | Late-stay / Weekend Call (Pulmonary, Nephrology, GI, Hematology) |
| PGY-1 | 26 | Male | Yes | In a relationship (cohabitating with partner) | No | CMG | Late-stay / Weekend Call (Pulmonary, Nephrology, GI, Hematology) |
| PGY-2 | 30 | Female | Yes | Single | Prefer not to answer | IMG | Late-stay / Weekend Call (Pulmonary, Nephrology, GI, Hematology) |
| PGY-2 | 27 | Male | No | Single | No | CMG | No call |
| PGY-3 | 31 | Female | Yes | Married | Yes | IMG | Home call |
| PGY-3 | 31 | Male | No | In a relationship (not cohabitating with partner) | No | CMG | Night Pool/Night Float |
| PGY-3 | 29 | Male | Yes | In a relationship (not cohabitating with partner) | No | CMG | No call |
| PGY-1 | 28 | Female | No | In a relationship (cohabitating with partner) | No | CMG | 1-in-4 In-house Call (CTU, CCU, ICU, etc) |
| PGY-3 | 30 | Male | Yes | Single | No | CMG | No call |
| PGY-2 | 28 | Female | No | Single | No | CMG | Late-stay / Weekend Call (Pulmonary, Nephrology, GI, Hematology) |
| PGY-1 | 32 | Male | Yes | Single | No | CMG | Home call |
| PGY-1 | 26 | Male | Yes | Married | No | CMG | Late-stay / Weekend Call (Pulmonary, Nephrology, GI, Hematology) |
| PGY-2 | 28 | Female | Yes | Single | No | CMG | No call |
| PGY-3 | 30 | Female | No | Married | No | CMG | No call |
| PGY-3 | 48 | Male | Yes | Married | Yes | IMG | Late-stay / Weekend Call (Pulmonary, Nephrology, GI, Hematology) |
| PGY-2 | 29 | Female | No | In a relationship (cohabitating with partner) | No | CMG | No call |
| PGY-2 | 29 | Male | Yes | Single | No | CMG | Late-stay / Weekend Call (Pulmonary, Nephrology, GI, Hematology) |
|  | 28 | Male | No | In a relationship (not cohabitating with partner) | No | CMG | 1-in-4 In-house Call (CTU, CCU, ICU, etc) |
| PGY-3 | 30 | Female | Yes | In a relationship (not cohabitating with partner) | No | CMG | Home call |
| PGY-3 | 27 | Female | Yes | In a relationship (not cohabitating with partner) | No | CMG | 1-in-4 In-house Call (CTU, CCU, ICU, etc) |
| PGY-3 | 28 | Female | Yes | In a relationship (cohabitating with partner) | No | CMG | Night Pool/Night Float |
| PGY-3 | 28 | Male | No | In a relationship (cohabitating with partner) | No | CMG | Night Pool/Night Float |
| PGY-3 | 34 | Female | Yes | Married | Yes | IMG | No call |
| PGY-2 | 35 | Female | No | Married | No | CMG | No call |
| PGY-2 |  | Prefer not to answer | Yes | Married | Yes | IMG | No call |
| PGY-3 | 28 | Female | Yes | Married | No | CMG | Night Pool/Night Float |
| PGY-1 | 26 | Male | No | In a relationship (cohabitating with partner) | No | CMG | Home call |
| PGY-2 | 33 | Male | No | In a relationship (not cohabitating with partner) | No | CMG | 1-in-4 In-house Call (CTU, CCU, ICU, etc) |
| PGY-3 | 26 | Male | No | In a relationship (cohabitating with partner) | No | CMG | 1-in-4 In-house Call (CTU, CCU, ICU, etc) |
| PGY-3 | 28 | Male | No | In a relationship (cohabitating with partner) | No | CMG | No call |
| PGY-1 | 28 | Female | No | In a relationship (cohabitating with partner) | No | CMG | 1-in-4 In-house Call (CTU, CCU, ICU, etc) |
| PGY-2 | 26 | Male | No | In a relationship (cohabitating with partner) | No | CMG | 1-in-4 In-house Call (CTU, CCU, ICU, etc) |
| PGY-2 | 28 | Female | No | Single | No | CMG | Night Pool/Night Float |
| PGY-1 | 27 | Male | Yes | Single | No | CMG | 1-in-4 In-house Call (CTU, CCU, ICU, etc) |
| PGY-1 | 31 | Female | No | Single | No | CMG | 1-in-4 In-house Call (CTU, CCU, ICU, etc) |
| PGY-2 | 27 | Female | Yes | In a relationship (cohabitating with partner) | No | CMG | Night Pool/Night Float |
| PGY-3 | 28 | Female | Yes | In a relationship (cohabitating with partner) | No | CMG | No call |
| PGY-3 | 28 | Female | Yes | Married | No | CMG | No call |
| PGY-2 |  | Female | Yes | Married | Yes | IMG | No call |
| PGY-1 | 25 | Female | Yes | In a relationship (not cohabitating with partner) | No | CMG | Home call |
| PGY-3 | 39 | Female | No | Married | Yes | IMG | Late-stay / Weekend Call (Pulmonary, Nephrology, GI, Hematology) |
| PGY-3 | 30 | Female | No | Married | No | CMG | Late-stay / Weekend Call (Pulmonary, Nephrology, GI, Hematology) |
| PGY-3 | 34 | Female | Yes | Married | Yes | CMG | Night Pool/Night Float |
| PGY-1 | 34 | Male | Yes | Married | Yes | IMG | 1-in-4 In-house Call (CTU, CCU, ICU, etc) |
| PGY-1 | 27 | Female | No | Single | No | CMG | 1-in-4 In-house Call (CTU, CCU, ICU, etc) |
| PGY-3 | 28 | Male | No | In a relationship (cohabitating with partner) | No | CMG | Night Pool/Night Float |
| PGY-3 | 31 | Male | No | In a relationship (not cohabitating with partner) | No | CMG | No call |
| PGY-3 | 31 | Female | Yes | Married | Yes | IMG | Late-stay / Weekend Call (Pulmonary, Nephrology, GI, Hematology) |
| PGY-2 | 29 | Male | Yes | Single | No | CMG | Late-stay / Weekend Call (Pulmonary, Nephrology, GI, Hematology) |
| PGY-1 |  | Male | Yes | Married | Yes | IMG | 1-in-4 In-house Call (CTU, CCU, ICU, etc) |
| PGY-2 | 27 | Female | No | In a relationship (not cohabitating with partner) | No | CMG | No call |
| PGY-3 | 33 | Female | Yes | Married | Yes | CMG | No call |
| PGY-2 | 25 | Female | Yes | In a relationship (cohabitating with partner) | No | CMG | No call |
| PGY-2 | 25 | Male | Yes | Single | No | CMG | Home call |
| PGY-2 | 29 | Female | No | In a relationship (cohabitating with partner) | No | CMG | Home call |
| PGY-1 | 28 | Male | No | Single | No | IMG | Late-stay / Weekend Call (Pulmonary, Nephrology, GI, Hematology) |
| PGY-1 | 27 | Male | Yes | In a relationship (not cohabitating with partner) | No | CMG | Home call |
| PGY-2 |  | Female | Yes | Married | Yes | IMG | 1-in-4 In-house Call (CTU, CCU, ICU, etc) |
| PGY-2 | 29 | Female | Yes | Single | No | CMG | Late-stay / Weekend Call (Pulmonary, Nephrology, GI, Hematology) |
| PGY-1 | 29 | Female | No | In a relationship (not cohabitating with partner) | No | CMG | 1-in-4 In-house Call (CTU, CCU, ICU, etc) |
| PGY-2 | 33 | Male | No | Married | Yes | CMG | 1-in-4 In-house Call (CTU, CCU, ICU, etc) |
| PGY-1 | 27 | Female | Yes | Single | No | IMG | 1-in-4 In-house Call (CTU, CCU, ICU, etc) |
| PGY-2 | 29 | Female | Yes | In a relationship (not cohabitating with partner) | No | CMG | No call |
| PGY-3 | 28 | Male | No | In a relationship (not cohabitating with partner) | No | CMG | Night Pool/Night Float |
| PGY-1 | 28 | Male | Yes | Single | No | CMG | No call |
| PGY-1 | 25 | Female | No | In a relationship (cohabitating with partner) | No | CMG | 1-in-4 In-house Call (CTU, CCU, ICU, etc) |
| PGY-2 | 27 | Male | Yes | In a relationship (cohabitating with partner) | No | CMG | Night Pool/Night Float |
| PGY-3 | 48 | Male | Yes | Married | Yes | IMG | Night Pool/Night Float |
| PGY-3 | 29 | Female | No | In a relationship (cohabitating with partner) | No | CMG | Home call |
| PGY-2 | 33 | Female | No | Prefer not to answer | No | CMG | No call |
| PGY-1 | 31 | Male | No | In a relationship (not cohabitating with partner) | No | CMG | 1-in-4 In-house Call (CTU, CCU, ICU, etc) |
| PGY-3 | 28 | Male | Yes | In a relationship (cohabitating with partner) | No | CMG | No call |
| PGY-1 | 27 | Male | Yes | In a relationship (not cohabitating with partner) | No | CMG | Late-stay / Weekend Call (Pulmonary, Nephrology, GI, Hematology) |
| PGY-2 | 28 | Female | No | Single | No | CMG | 1-in-4 In-house Call (CTU, CCU, ICU, etc) |
| PGY-3 | 31 | Female | Yes | Married | Yes | IMG | Late-stay / Weekend Call (Pulmonary, Nephrology, GI, Hematology) |
| PGY-1 | 26 | Female | Yes | In a relationship (cohabitating with partner) | No | CMG | Late-stay / Weekend Call (Pulmonary, Nephrology, GI, Hematology) |
| PGY-2 | 27 | Female | No | Married | No | CMG | Night Pool/Night Float |
| PGY-3 | 28 | Male | Yes | In a relationship (cohabitating with partner) | No | CMG | Late-stay / Weekend Call (Pulmonary, Nephrology, GI, Hematology) |
| PGY-2 |  | Prefer not to answer | Yes | Married | Yes | IMG | Night Pool/Night Float |
| PGY-3 | 32 | Female | Yes | In a relationship (cohabitating with partner) | No | CMG | No call |
| PGY-2 | 27 | Male | No | In a relationship (cohabitating with partner) | No | CMG | Night Pool/Night Float |
| PGY-3 | 31 | Female | Yes | Married | Yes | IMG | Night Pool/Night Float |
| PGY-2 | 29 | Female | Yes | In a relationship (not cohabitating with partner) | No | CMG | No call |
| PGY-3 | 28 | Male | No | In a relationship (cohabitating with partner) | No | CMG | No call |
| PGY-1 | 25 | Female | Yes | In a relationship (not cohabitating with partner) | No | CMG | Late-stay / Weekend Call (Pulmonary, Nephrology, GI, Hematology) |
| PGY-3 | 30 | Female | No | Married | No | CMG | Home call |
| PGY-3 | 28 | Female | Yes | In a relationship (cohabitating with partner) | No | CMG | No call |
| PGY-1 | 27 | Male | Yes | In a relationship (not cohabitating with partner) | No | CMG | 1-in-4 In-house Call (CTU, CCU, ICU, etc) |
| PGY-1 | 27 | Female | No | Single | No | CMG | Late-stay / Weekend Call (Pulmonary, Nephrology, GI, Hematology) |
| PGY-3 | 28 | Male | No | In a relationship (cohabitating with partner) | No | CMG | No call |
| PGY-2 | 28 | Male | Yes | In a relationship (cohabitating with partner) | No | CMG | No call |
| PGY-3 | 48 | Male | Yes | Married | Yes | IMG | No call |
| PGY-2 | 27 | Female | Yes | In a relationship (cohabitating with partner) | No | CMG | Home call |
| PGY-1 | 34 | Male | Yes | Married | Yes | IMG | Late-stay / Weekend Call (Pulmonary, Nephrology, GI, Hematology) |
| PGY-2 | 29 | Female | No | In a relationship (cohabitating with partner) | No | CMG | Late-stay / Weekend Call (Pulmonary, Nephrology, GI, Hematology) |
| PGY-1 | 27 | Male | Yes | In a relationship (not cohabitating with partner) | No | CMG | No call |
| PGY-2 | 28 | Female | No | Single | No | CMG | 1-in-4 In-house Call (CTU, CCU, ICU, etc) |
| PGY-2 |  | Female | Yes | In a relationship (cohabitating with partner) | No | IMG | Home call |
| PGY-1 | 26 | Male | No | In a relationship (cohabitating with partner) | No | CMG | Late-stay / Weekend Call (Pulmonary, Nephrology, GI, Hematology) |
| PGY-1 | 28 | Female | No | In a relationship (cohabitating with partner) | No | CMG | Late-stay / Weekend Call (Pulmonary, Nephrology, GI, Hematology) |
| PGY-3 | 34 | Female | Yes | Married | Yes | IMG | No call |
| PGY-2 | 25 | Male | Yes | In a relationship (not cohabitating with partner) | No | CMG | Late-stay / Weekend Call (Pulmonary, Nephrology, GI, Hematology) |
| PGY-3 | 27 | Female | Yes | In a relationship (not cohabitating with partner) | No | CMG | No call |
| PGY-3 | 33 | Female | Yes | Married | Yes | CMG | No call |
| PGY-2 | 35 | Female | No | Married | No | CMG | Home call |
| PGY-3 | 27 | Male | No | In a relationship (cohabitating with partner) | No | CMG | Late-stay / Weekend Call (Pulmonary, Nephrology, GI, Hematology) |
| PGY-1 | 26 | Female | Yes | Single | No | IMG | Late-stay / Weekend Call (Pulmonary, Nephrology, GI, Hematology) |
| PGY-1 | 29 | Female | No | In a relationship (not cohabitating with partner) | No | CMG | No call |
| PGY-1 | 28 | Male | Yes | In a relationship (cohabitating with partner) | No | CMG | 1-in-4 In-house Call (CTU, CCU, ICU, etc) |
| PGY-1 | 28 | Female | Yes | Married | Yes | CMG | 1-in-4 In-house Call (CTU, CCU, ICU, etc) |
| PGY-3 | 29 | Female | No | In a relationship (cohabitating with partner) | No | CMG | No call |
| PGY-2 | 28 | Female | No | Single | No | CMG | Home call |
| PGY-1 |  | Male |  | Married | Yes | IMG | Late-stay / Weekend Call (Pulmonary, Nephrology, GI, Hematology) |
| PGY-2 |  | Female | Yes | Married | Yes | IMG | 1-in-4 In-house Call (CTU, CCU, ICU, etc) |
| PGY-1 | 28 | Male | No | Single | No | IMG | Night Pool/Night Float |
| PGY-1 | 27 | Male | Yes | Single | No | CMG | 1-in-4 In-house Call (CTU, CCU, ICU, etc) |
| PGY-1 | 28 | Male | Yes | In a relationship (not cohabitating with partner) | No | CMG | 1-in-4 In-house Call (CTU, CCU, ICU, etc) |
| PGY-3 | 29 | Male | Yes | In a relationship (not cohabitating with partner) | No | CMG | No call |
| PGY-1 | 38 | Female | No | Married | Yes | IMG | Late-stay / Weekend Call (Pulmonary, Nephrology, GI, Hematology) |
| PGY-3 | 28 | Male | No | In a relationship (not cohabitating with partner) | No | CMG | No call |
| PGY-3 | 39 | Female | No | Married | Yes | IMG | Late-stay / Weekend Call (Pulmonary, Nephrology, GI, Hematology) |

**Supplemental Table 2**. Burnout data

| **Describe how often, if ever, you ever feel this way about your job: [I feel emotionally drained from my work]** | **Describe how often, if ever, you ever feel this way about your job: [I feel used up at the end of the workday]** | **Describe how often, if ever, you ever feel this way about your job: [I feel fatigued when I get up in the morning and have to face another day on the job. ]** | **Describe how often, if ever, you ever feel this way about your job: [I can easily understand how my patients feel about things.]** | **Describe how often, if ever, you ever feel this way about your job: [I feel I treat some patients as if they were impersonal objects]** | **Describe how often, if ever, you ever feel this way about your job: [Working with people all day is really a strain for me.]** | **Describe how often, if ever, you ever feel this way about your job: [I deal very effectively with the problems of my patients.]** | **Describe how often, if ever, you ever feel this way about your job: [I feel burned out from my work.]** | **Describe how often, if ever, you ever feel this way about your job: [I feel I'm positively influencing other people's lives through my work.]** | **Describe how often, if ever, you ever feel this way about your job: [I've become more callous toward people since I took this job.]** | **Describe how often, if ever, you ever feel this way about your job: [I worry that this job is hardening me emotionally.]** | **Describe how often, if ever, you ever feel this way about your job: [I feel very energetic.]** | **Describe how often, if ever, you ever feel this way about your job: [I feel frustrated by my job.]** | **Describe how often, if ever, you ever feel this way about your job: [I feel I'm working too hard on my job.]** | **Describe how often, if ever, you ever feel this way about your job: [I don't really care what happens to some patients.]** | **Describe how often, if ever, you ever feel this way about your job: [Working with people directly puts too much stress on me.]** | **Describe how often, if ever, you ever feel this way about your job: [I can easily create a relaxed atmosphere with my patients.]** | **Describe how often, if ever, you ever feel this way about your job: [I feel exhilarated after working closely with my patients.]** | **Describe how often, if ever, you ever feel this way about your job: [I have accomplished many worthwhile things in this job.]** | **Describe how often, if ever, you ever feel this way about your job: [I feel like I'm at the end of my rope.]** | **Describe how often, if ever, you ever feel this way about your job: [In my work, I deal with emotional problems very calmly.]** | **Describe how often, if ever, you ever feel this way about your job: [I feel patients blame me for some of their problems.]** |
| --- | --- | --- | --- | --- | --- | --- | --- | --- | --- | --- | --- | --- | --- | --- | --- | --- | --- | --- | --- | --- | --- |
| 5 | 5 | 4 | 6 | 0 | 1 | 4 | 1 | 6 | 1 | 0 | 3 | 1 | 6 | 0 | 2 | 6 | 6 | 6 | 4 | 5 | 4 |
| 4 | 3 | 5 | 6 | 3 | 3 | 5 | 2 | 4 | 1 | 5 | 5 | 3 | 4 | 0 | 2 | 6 | 2 | 4 | 1 | 6 | 1 |
| 5 | 5 | 2 | 2 | 1 | 1 | 5 | 2 | 5 | 3 | 3 | 4 | 1 | 1 | 1 | 1 | 5 | 4 | 4 | 0 | 5 | 0 |
| 3 | 4 | 4 | 5 | 2 | 0 | 6 | 3 | 5 | 5 | 2 | 5 | 4 | 4 | 0 | 0 | 6 | 5 | 4 | 1 | 0 | 1 |
| 4 | 5 | 5 | 6 | 3 | 0 | 5 | 3 | 5 | 0 | 0 | 4 |  | 3 | 2 | 0 | 5 | 5 | 5 | 0 | 6 | 1 |
| 1 | 2 | 2 | 5 | 5 | 3 | 5 | 1 | 4 | 5 | 4 | 3 | 3 | 4 | 2 | 2 | 4 | 3 | 4 | 1 | 3 | 3 |
| 4 | 4 | 5 | 6 | 0 | 0 | 4 | 2 | 5 | 2 | 1 | 2 | 3 | 3 | 0 | 3 | 5 | 4 | 3 | 3 | 3 | 2 |
| 5 | 4 | 3 | 1 | 1 | 1 | 4 | 4 | 5 | 0 | 3 | 3 | 1 | 4 | 0 | 0 | 5 | 4 | 5 | 0 | 5 | 1 |
| 2 | 2 | 3 | 5 | 0 | 0 | 4 | 2 | 6 | 1 | 1 | 5 | 1 | 4 | 0 | 0 | 4 | 5 | 6 | 0 | 1 | 3 |
| 5 | 6 | 4 | 6 | 2 | 4 | 6 | 5 | 4 | 4 | 6 | 1 | 5 | 6 | 1 | 3 | 6 | 1 | 0 | 5 | 5 | 1 |
| 5 | 5 | 5 | 5 | 3 | 4 | 5 | 4 | 6 | 2 | 2 | 3 | 3 | 3 | 2 | 1 | 3 | 1 | 3 | 1 | 6 | 1 |
| 2 | 3 | 3 | 2 | 1 | 1 | 5 | 3 | 1 | 0 | 5 | 4 | 0 | 0 | 0 | 1 | 3 |  | 1 | 1 | 6 | 1 |
| 1 | 1 | 2 | 2 | 2 | 1 | 2 | 2 | 1 | 1 | 2 | 2 | 2 | 2 | 2 | 1 | 2 | 2 | 1 | 1 | 1 | 1 |
| 4 | 4 | 5 | 6 | 4 | 4 | 5 | 4 | 5 | 4 | 4 | 3 | 2 | 2 | 2 | 3 | 5 | 5 | 5 | 2 | 5 | 4 |
| 5 | 5 | 5 | 6 | 3 | 4 | 5 | 4 | 4 | 5 | 2 | 4 | 6 | 6 | 5 | 4 | 5 | 3 | 5 | 1 | 6 | 3 |
| 5 | 5 | 5 | 6 | 3 | 2 | 5 | 5 | 5 | 2 | 5 | 4 | 4 | 3 | 0 | 1 | 4 | 5 | 5 | 4 | 5 | 1 |
| 5 | 5 | 3 | 6 | 0 | 0 | 6 | 1 | 6 | 0 | 5 | 5 | 4 | 5 | 1 | 0 | 6 | 6 | 6 | 0 | 4 | 5 |
| 3 | 4 | 5 | 6 | 1 | 2 | 5 | 3 | 5 | 2 | 6 | 4 | 4 | 6 | 1 | 1 | 6 | 5 | 5 | 1 | 5 | 1 |
| 4 | 4 | 4 | 5 | 1 | 5 | 5 | 5 | 3 | 3 | 5 | 1 | 4 | 3 | 0 | 4 | 1 | 1 | 1 | 4 | 4 | 2 |
| 2 | 2 | 1 | 1 | 0 | 0 | 3 | 1 | 3 | 0 | 0 | 2 | 0 | 2 | 0 | 0 | 3 | 0 | 3 | 0 | 3 | 0 |
| 4 | 6 | 5 | 5 | 4 | 5 | 6 | 5 | 5 | 4 | 4 | 3 | 4 | 5 | 4 | 3 | 5 | 4 | 5 | 3 | 5 | 3 |
| 3 | 3 | 4 | 5 | 0 | 1 | 5 | 4 | 4 | 1 | 1 | 2 | 2 | 5 | 0 | 1 | 5 | 1 | 1 | 2 | 5 | 0 |
| 3 | 6 | 6 | 5 | 2 | 3 | 2 | 5 | 6 | 0 | 2 | 1 | 0 | 2 | 0 | 0 | 4 | 5 | 0 | 4 | 5 | 0 |
| 1 | 2 | 1 | 3 | 0 | 0 | 6 | 1 | 5 | 0 | 1 | 4 | 1 | 0 | 0 | 1 | 6 | 6 | 6 | 0 | 5 | 1 |
| 5 | 6 | 4 | 6 | 0 | 0 |  | 5 | 3 | 2 | 3 | 2 | 2 | 1 | 0 | 1 | 6 | 4 | 3 | 3 | 4 | 0 |
| 4 | 5 | 5 | 5 | 4 | 4 | 4 | 5 | 5 | 4 | 4 | 4 | 5 | 5 | 4 | 4 | 4 | 3 | 3 | 3 | 4 | 3 |
| 5 | 5 | 4 | 3 | 4 | 4 | 5 | 5 | 4 | 5 | 4 | 5 | 5 | 5 | 6 | 4 | 6 | 4 | 4 | 3 | 4 | 5 |
| 5 | 5 | 5 | 2 | 4 | 4 | 3 | 6 | 2 | 6 | 6 | 2 | 5 | 2 | 6 | 2 | 3 | 2 | 2 | 5 | 2 | 3 |
| 3 | 4 | 3 | 5 | 0 | 1 | 5 | 5 | 5 | 0 | 1 | 4 | 4 | 4 | 0 | 1 | 5 | 5 | 6 | 1 | 6 | 3 |
| 4 | 5 | 5 | 6 | 1 | 2 | 6 | 3 | 6 | 0 | 1 | 5 | 5 | 5 | 0 | 0 | 6 | 5 | 5 | 1 | 6 | 1 |
| 1 | 2 | 2 | 3 | 0 | 1 | 2 | 1 | 3 | 0 | 0 | 1 | 1 | 0 | 0 | 1 | 3 | 3 | 3 | 0 | 3 | 0 |
| 5 | 5 | 5 | 5 | 1 | 4 | 2 | 4 | 2 | 0 | 0 | 1 | 2 | 2 | 0 | 2 | 4 | 3 | 2 | 3 | 5 | 2 |
| 0 | 3 | 1 | 5 | 0 | 1 | 5 | 0 | 6 | 0 | 0 | 4 | 0 | 0 | 0 | 0 | 6 | 0 | 6 | 0 | 6 | 0 |
| 5 | 5 | 3 | 6 | 1 | 1 | 5 | 5 | 5 | 1 | 2 | 3 | 3 | 2 | 0 | 0 | 5 | 5 | 5 | 2 | 5 | 1 |
| 4 | 3 | 3 | 6 | 3 | 0 | 6 | 3 | 6 | 1 | 1 | 5 | 1 | 2 | 0 | 0 | 6 | 6 | 6 | 0 | 6 | 2 |
| 5 | 3 | 4 | 5 | 4 | 4 | 6 | 3 | 4 | 6 | 6 | 5 | 5 | 3 | 3 | 3 | 5 | 5 | 6 | 1 | 5 | 2 |
| 0 | 1 | 1 | 2 | 5 | 0 | 5 | 1 | 6 | 0 | 6 | 6 | 1 | 1 | 5 | 0 | 6 | 2 | 6 | 1 | 6 | 1 |
| 6 | 3 | 1 | 1 | 0 | 1 | 1 | 3 | 2 | 3 | 1 | 3 | 2 | 3 | 3 | 2 | 4 | 2 | 4 | 1 | 6 | 0 |
| 3 | 4 | 3 | 5 | 3 | 2 | 5 | 2 | 5 | 3 | 2 | 5 | 2 | 2 | 2 | 2 | 5 | 5 | 5 | 1 | 5 | 3 |
| 4 | 4 | 2 | 6 | 0 | 0 | 6 | 4 | 5 | 0 | 1 | 5 | 0 | 4 | 0 | 0 | 6 | 5 | 5 | 0 | 6 | 1 |
| 1 | 2 | 1 | 6 | 1 | 1 | 5 | 1 | 5 | 3 | 2 | 5 | 1 | 0 | 0 | 1 | 6 | 5 | 3 | 0 | 6 | 0 |
| 1 | 1 | 0 | 2 | 0 | 0 | 2 | 0 | 2 | 0 | 0 | 2 | 0 | 1 | 0 | 0 | 2 | 2 | 2 | 0 | 2 | 0 |
| 5 | 5 | 5 | 4 | 5 | 2 | 3 | 5 | 4 | 5 | 4 | 2 | 1 | 3 | 1 | 2 | 4 | 4 | 4 | 0 | 3 | 4 |
| 5 | 6 | 6 | 6 | 3 | 4 | 5 | 2 | 4 | 5 | 4 | 4 | 5 | 5 | 5 | 4 | 5 | 3 | 6 | 2 | 4 | 6 |
| 0 | 0 | 1 | 3 | 0 | 0 | 2 | 0 | 2 | 1 | 0 | 3 | 0 | 3 | 0 | 1 | 3 | 0 | 2 | 0 | 3 | 0 |
| 2 | 3 | 4 | 6 | 2 | 1 | 4 | 2 | 5 | 1 | 3 | 5 | 2 | 2 | 1 | 1 | 4 | 5 | 5 | 1 | 5 | 2 |
| 4 | 5 | 5 | 6 | 0 | 0 | 6 | 3 | 6 | 0 | 0 | 4 | 0 | 4 | 0 | 3 | 5 | 5 | 5 | 0 | 3 | 1 |
| 3 | 5 | 3 | 5 | 2 | 4 | 5 | 3 | 2 | 3 | 4 | 5 | 3 | 3 | 2 | 2 | 5 | 2 | 2 | 1 | 4 | 5 |
| 0 | 0 | 3 | 6 | 1 | 0 | 1 | 1 | 0 | 6 | 6 | 2 | 1 | 2 | 0 | 2 | 6 | 0 | 5 | 1 | 6 | 0 |
| 4 | 4 | 3 | 6 | 2 | 1 | 5 | 3 | 4 | 3 | 4 | 3 | 4 | 4 | 2 | 1 | 5 | 3 | 3 | 1 | 5 | 2 |
| 4 | 4 | 4 | 4 | 1 | 3 | 6 | 4 | 4 | 3 | 2 | 4 | 3 | 5 | 0 | 1 | 5 | 3 | 3 | 1 | 6 | 1 |
| 4 | 4 | 3 | 5 | 2 | 2 | 5 | 3 | 5 | 2 | 4 | 4 | 3 | 3 | 0 | 3 | 6 | 4 | 5 | 1 | 5 | 4 |
| 1 | 1 | 1 | 6 | 0 | 0 | 6 | 1 | 6 | 1 | 1 | 5 | 1 | 1 | 0 | 0 | 6 | 5 | 5 | 0 | 6 | 0 |
| 5 | 5 | 5 | 6 | 1 | 2 | 5 | 4 | 5 | 2 | 3 | 3 | 4 | 5 | 0 | 1 | 5 | 5 | 5 | 3 | 5 | 4 |
| 4 | 6 | 5 | 6 | 5 | 3 | 5 | 3 | 5 | 0 | 6 | 5 | 2 | 1 | 0 | 0 | 5 | 5 | 5 | 3 | 5 | 2 |
| 5 | 5 | 4 | 5 | 4 | 3 | 5 | 2 | 5 | 0 | 1 | 5 | 4 | 4 | 0 | 2 | 5 | 4 | 4 | 0 | 5 | 0 |
| 6 | 6 | 6 | 5 | 4 | 5 | 5 | 6 | 5 | 5 | 5 | 3 | 5 | 5 | 3 | 5 | 5 | 3 | 4 | 3 | 5 | 5 |
| 6 | 6 | 6 | 6 | 4 | 5 | 5 | 6 | 5 | 5 | 5 | 3 | 5 | 5 | 4 | 2 | 5 | 5 | 5 | 4 | 5 | 4 |
| 1 | 1 | 0 | 5 | 0 | 0 | 6 | 0 | 5 | 0 | 0 | 6 | 1 | 1 | 0 | 0 | 6 | 6 | 5 | 0 | 5 | 1 |
| 6 | 6 | 6 | 4 | 0 | 5 | 4 | 6 | 2 | 5 | 6 | 0 | 6 | 6 | 3 | 6 | 5 | 0 | 0 | 6 | 2 | 4 |
| 0 | 1 | 1 | 5 | 0 | 0 | 6 | 1 | 6 | 0 | 1 | 5 | 1 | 4 | 0 | 0 | 4 | 4 | 6 | 0 | 6 | 3 |
| 0 | 1 | 1 | 2 | 0 | 0 | 2 | 2 | 1 | 0 | 2 | 2 | 1 | 1 | 0 | 0 | 1 | 1 | 2 | 0 | 2 | 1 |
| 4 | 5 | 2 | 1 | 1 | 1 | 5 | 1 | 6 | 0 | 1 | 3 | 5 | 2 | 1 | 1 | 5 | 4 | 5 | 1 | 6 | 5 |
| 5 | 4 | 5 | 5 | 3 | 3 | 5 | 5 | 5 | 6 | 5 | 4 | 6 | 6 | 4 | 4 | 6 | 5 | 5 | 4 | 6 | 4 |
| 3 | 5 | 5 | 6 | 0 | 2 | 5 | 1 | 6 | 2 | 6 | 3 | 2 | 5 | 0 | 1 | 6 | 5 | 6 | 1 | 6 | 2 |
| 5 | 5 | 5 | 6 | 3 | 2 | 5 | 5 | 4 | 4 | 2 | 4 | 5 | 5 | 1 | 4 | 5 | 5 | 4 | 0 | 5 | 1 |
| 4 | 4 | 4 | 5 | 0 | 1 | 6 | 3 | 2 | 1 | 5 | 1 | 2 | 5 | 0 | 2 | 5 | 2 | 2 | 1 | 5 | 0 |
| 2 | 3 | 5 | 6 | 1 | 3 | 5 | 3 | 1 | 2 | 2 | 2 | 1 | 2 | 0 | 1 | 5 | 5 | 4 | 4 | 4 | 3 |
| 3 | 4 | 1 | 6 | 0 | 1 | 6 | 1 | 6 | 0 | 0 | 4 | 1 | 1 | 0 | 0 | 6 | 5 | 5 | 2 | 1 | 4 |
| 2 | 4 | 3 | 3 | 0 | 0 | 5 | 3 | 6 | 1 | 2 | 4 | 3 | 3 | 0 | 0 | 3 | 5 | 5 | 3 | 5 | 2 |

**Supplemental Table 3**. Fatigue data

| Over the current block, please describe to what extent you have experienced the following, from 1 being none, and 10 being maximal: [Physical Exertion (warm, sweaty, breathing heavily, etc)] | Over the current block, please describe to what extent you have experienced the following, from 1 being none, and 10 being maximal: [Physical Discomfort (aching, tense muscles, stiff joints, etc)] | Over the current block, please describe to what extent you have experienced the following, from 1 being none, and 10 being maximal: [Lack of Motivation] | Over the current block, please describe to what extent you have experienced the following, from 1 being none, and 10 being maximal: [Sleepiness] | Over the current block, please describe to what extent you have experienced the following, from 1 being none, and 10 being maximal: [Lack of Energy] |
| --- | --- | --- | --- | --- |
| 5 | 5 | 5 | 7 | 7 |
| 1 | 2 | 3 | 4 | 3 |
| 1 | 3 | 5 | 3 | 5 |
| 1 | 1 | 1 | 9 | 2 |
| 2 | 2 | 1 | 5 | 5 |
| 1 | 1 | 3 | 2 | 2 |
| 6 | 2 | 3 | 5 | 5 |
| 1 | 3 | 3 | 8 | 8 |
| 1 | 1 | 2 | 4 | 3 |
| 3 | 5 | 5 | 5 | 5 |
| 4 | 6 | 5 | 6 | 6 |
| 3 | 3 | 8 | 3 | 4 |
| 6 | 7 | 4 | 4 | 4 |
| 6 | 6 | 6 | 8 | 8 |
| 2 | 2 | 6 | 7 | 7 |
| 3 | 6 | 2 | 7 | 5 |
| 4 | 6 | 5 | 7 | 7 |
| 1 | 1 | 6 | 9 | 7 |
| 5 | 5 | 9 | 8 | 8 |
| 2 | 5 | 2 | 6 | 6 |
| 7 | 6 | 9 | 10 | 9 |
| 3 | 4 | 6 | 6 | 7 |
| 8 | 9 | 5 | 9 | 9 |
| 1 | 1 | 1 | 3 | 1 |
| 3 | 2 | 4 | 2 | 3 |
| 2 | 8 | 8 | 9 | 9 |
| 1 | 2 | 3 | 3 | 4 |
| 7 | 9 | 9 | 9 | 9 |
| 1 | 1 | 2 | 2 | 2 |
| 2 | 2 | 5 | 2 | 6 |
| 1 | 1 | 1 | 1 | 1 |
| 5 | 8 | 6 | 7 | 6 |
| 1 | 1 | 1 | 1 | 1 |
| 2 | 7 | 4 | 5 | 5 |
| 7 | 2 | 1 | 5 | 3 |
| 2 | 3 | 3 | 5 | 5 |
| 1 | 2 | 2 | 8 | 2 |
| 4 | 4 | 10 | 7 | 7 |
| 2 | 3 | 3 | 6 | 6 |
| 2 | 1 | 2 | 2 | 2 |
| 2 | 5 | 2 | 5 | 4 |
| 2 | 2 | 2 | 3 | 3 |
| 3 | 7 | 7 | 8 | 8 |
| 8 | 8 | 5 | 5 | 7 |
| 1 | 2 | 1 | 2 | 2 |
| 3 | 3 | 4 | 5 | 5 |
| 8 | 7 | 1 | 3 | 3 |
| 2 | 4 | 7 | 9 | 9 |
| 2 | 4 | 5 | 4 | 5 |
| 7 | 4 | 8 | 6 | 5 |
| 1 | 4 | 3 | 8 | 6 |
| 3 | 8 | 3 | 7 | 5 |
| 2 | 1 | 1 | 3 | 3 |
| 2 | 5 | 5 | 7 | 7 |
| 6 | 6 | 4 | 6 | 7 |
| 4 | 2 | 3 | 5 | 6 |
| 2 | 7 | 8 | 5 | 8 |
| 9 | 9 | 9 | 9 | 9 |
| 1 | 1 | 2 | 3 | 1 |
| 8 | 8 | 9 | 9 | 9 |
| 1 | 3 | 1 | 4 | 3 |
| 6 | 7 | 5 | 7 | 5 |
| 2 | 4 | 1 | 3 | 3 |
| 1 | 1 | 3 | 3 | 3 |
| 1 | 1 | 5 | 8 | 7 |
| 5 | 2 | 5 | 8 | 8 |
| 4 | 5 | 5 | 6 | 6 |
| 6 | 2 | 6 | 6 | 5 |
| 1 | 2 | 2 | 2 | 3 |
| 1 | 2 | 2 | 3 | 2 |
| 2 | 3 | 1 | 3 | 3 |
| 3 | 1 | 6 | 8 | 5 |
| 5 | 5 | 5 | 9 | 5 |
| 5 | 2 | 2 | 3 | 2 |
| 3 | 8 | 7 | 8 | 8 |
| 1 | 5 | 3 | 1 | 3 |
| 6 | 8 | 8 | 9 | 9 |
| 7 | 7 | 7 | 8 | 8 |
| 3 | 3 | 3 | 3 | 4 |
| 7 | 7 | 8 | 8 | 8 |
| 1 | 1 | 3 | 4 | 2 |
| 1 | 1 | 1 | 2 | 2 |
| 1 | 2 | 2 | 2 | 2 |
| 5 | 7 | 4 | 6 | 6 |
| 3 | 3 | 2 | 3 | 4 |
| 5 | 8 | 7 | 10 | 10 |
| 3 | 1 | 8 | 8 | 8 |
| 5 | 5 | 3 | 3 | 3 |
| 1 | 1 | 3 | 3 | 3 |
| 5 | 5 | 4 | 6 | 5 |
| 5 | 6 | 8 | 8 | 8 |
| 9 | 10 | 4 | 8 | 8 |
| 1 | 1 | 1 | 1 | 1 |
| 1 | 1 | 1 | 1 | 1 |
| 4 | 6 | 6 | 5 | 5 |
| 2 | 3 | 3 | 3 | 3 |
| 9 | 8 | 2 | 2 | 2 |
| 3 | 3 | 3 | 3 | 2 |
| 8 | 8 | 10 | 10 | 10 |
| 4 | 7 | 6 | 6 | 6 |
| 3 | 3 | 1 | 6 | 5 |
| 2 | 2 | 1 | 2 | 2 |
| 1 | 2 | 4 | 4 | 4 |
| 6 | 5 | 7 | 8 | 6 |
| 6 | 8 | 5 | 6 | 4 |
| 9 | 9 | 8 | 8 | 8 |
| 1 | 1 | 3 | 3 | 1 |
| 2 | 6 | 6 | 6 | 6 |
| 1 | 1 | 1 | 1 | 1 |
| 2 | 6 | 3 | 6 | 3 |
| 3 | 3 | 6 | 5 | 3 |
| 1 | 1 | 1 | 1 | 1 |
| 2 | 6 | 6 | 7 | 6 |
| 6 | 9 | 6 | 10 | 10 |
| 5 | 7 | 5 | 7 | 7 |
| 5 | 5 | 6 | 8 | 8 |
| 3 | 3 | 4 | 6 | 6 |
| 6 | 6 | 6 | 8 | 8 |
| 1 | 8 | 5 | 7 | 9 |
| 6 | 4 | 5 | 5 | 5 |
| 2 | 5 | 4 | 5 | 5 |
| 5 | 5 | 8 | 9 | 9 |
| 6 | 8 | 3 | 9 | 9 |
| 1 | 1 | 7 | 1 | 1 |
| 2 | 2 | 5 | 8 | 8 |
| 4 | 4 | 2 | 4 | 4 |
| 1 | 1 | 1 | 1 | 1 |
| 5 | 5 | 4 | 6 | 5 |
| 1 | 1 | 2 | 2 | 2 |
| 3 | 5 | 4 | 5 | 4 |
| 1 | 1 | 8 | 9 | 9 |
| 3 | 3 | 4 | 4 | 5 |
| 1 | 1 | 2 | 2 | 2 |
| 1 | 1 | 1 | 5 | 5 |
| 6 | 6 | 7 | 7 | 7 |
| 1 | 1 | 1 | 1 | 1 |
| 7 | 5 | 5 | 5 | 6 |
| 2 | 2 | 2 | 3 | 2 |
| 5 | 6 | 7 | 7 | 7 |
| 2 | 2 | 2 | 2 | 2 |
| 5 | 7 | 7 | 8 | 8 |
| 2 | 2 | 2 | 2 | 2 |
| 3 | 3 | 2 | 3 | 3 |
| 2 | 7 | 2 | 3 | 2 |
| 3 | 9 | 4 | 5 | 7 |
| 5 | 6 | 4 | 7 | 6 |
| 1 | 1 | 4 | 4 | 4 |
| 5 | 6 | 6 | 7 | 8 |
| 3 | 3 | 2 | 2 | 1 |
| 3 | 3 | 3 | 5 | 4 |
| 9 | 10 | 9 | 7 | 8 |
| 2 | 2 | 2 | 5 | 2 |
| 7 | 8 | 6 | 8 | 8 |
| 5 | 8 | 6 | 8 | 9 |
| 1 | 2 | 1 | 1 | 2 |
| 6 | 7 | 9 | 9 | 9 |
| 1 | 1 | 1 | 1 | 1 |
| 4 | 4 | 4 | 2 | 3 |
| 1 | 1 | 1 | 1 | 1 |
| 7 | 8 | 7 | 8 | 8 |
| 2 | 3 | 6 | 4 | 8 |
| 1 | 1 | 1 | 1 | 1 |
| 3 | 7 | 1 | 2 | 4 |
| 1 | 1 | 1 | 1 | 1 |
| 6 | 7 | 7 | 8 | 7 |

**Supplemental Table 4**. Sleep and impacts of fatigue data

| **On average, how many hours of sleep did you have, per call shift (if applicable)?** | **On average, how many hours of sleep per day did you have between work days, while not on call?** | **During this rotation patient care was directly and negatively affected as a result of medical error(s) / negligence (eg. medication incorrectly dosed, unstable patient not sent to observation unit)** | **During this rotation, I did things that were unprofessional, not collegial, or negatively impacted the dynamics of the healthcare team (eg. calling consultants late, discharge summary dictated late, argument with nursing).** | **During this rotation fatigue put me at significant risk of personal harm (unsafe driving/cycling, needle-stick injuries, other occupational injuries, or household hazards such as leaving doors unlocked or the stove on)** | **During this rotation, fatigue put me at significant risk of unprofessionalism or negatively impacting relationships? (irritability with colleagues, irritability over the phone, negative impacts on relationships with friends/family due to fatigue)** | **During the block, how often did fatigue impact the management of your own personal health or medical conditions? (Personal condition management on call, medication adherence, etc)** |
| --- | --- | --- | --- | --- | --- | --- |
|  |  | 1 | 1 | 1 | 1 | 3 |
| 0 | 7 | 1 | 1 | 2 | 3 | 2 |
| 3.5 | 7 | 2 | 1 | 1 | 2 | 1 |
|  | 7 | 2 | 2 | 1 | 3 | 2 |
| 2.5 | 6 | 3 | 1 | 3 | 4 | 4 |
| 7 | 7 | 1 | 1 | 1 | 1 | 1 |
| 0 | 6.5 | 1 | 1 | 1 | 2 | 3 |
| 2.5 | 6.5 | 3 | 2 | 2 | 2 | 2 |
| 6.5 | 8 | 1 | 1 | 1 | 1 | 1 |
|  | 8 | 1 | 1 | 2 | 3 | 4 |
| 7.5 | 7.5 | 1 | 1 | 1 | 2 | 2 |
|  | 6 | 2 | 1 | 4 | 1 | 3 |
| 5 | 11 | 4 | 2 | 2 | 2 | 5 |
| 1 | 6 | 2 | 2 | 3 | 1 | 4 |
|  | 7 | 2 | 2 | 1 | 3 | 2 |
| 0.5 | 5.5 | 1 | 1 | 4 | 5 | 4 |
| 0.5 | 8 | 1 | 1 | 3 | 2 | 2 |
|  | 7.5 | 1 | 1 | 1 | 2 | 4 |
|  |  | 3 | 2 | 4 | 2 | 5 |
| 0.5 |  | 2 | 1 | 2 | 3 | 5 |
|  | 5 | 1 | 2 | 3 | 4 | 4 |
|  | 6 | 1 | 1 | 1 | 3 | 2 |
| 0 | 5.5 | 1 | 1 | 2 | 4 | 3 |
| 0 | 9 | 1 | 1 | 2 | 1 | 1 |
| 2 | 5 | 1 | 1 | 2 | 2 | 3 |
| 4 | 7 | 2 | 1 | 2 | 3 | 5 |
|  | 7 | 1 | 1 | 1 | 2 | 1 |
| 2 |  | 1 | 1 | 2 | 4 | 4 |
| 7 | 7 | 1 | 1 | 1 | 1 | 3 |
| 0 | 6 | 1 | 1 | 1 | 1 | 1 |
| 1 | 7 | 1 | 1 | 1 | 1 | 1 |
|  | 5 | 2 | 2 | 3 | 4 | 4 |
| 0 | 7 | 1 | 1 | 1 | 1 | 1 |
|  | 6.5 | 1 | 1 | 1 | 3 | 2 |
|  | 7 | 1 | 1 | 1 | 1 | 1 |
|  | 7 | 1 | 2 | 2 | 3 | 1 |
| 0.5 | 6 | 1 | 1 | 1 | 1 | 1 |
| 3 | 5.5 | 1 | 1 | 2 | 1 | 4 |
| 2.5 | 6 | 5 | 1 | 3 | 4 | 3 |
|  | 6 | 1 | 1 | 1 | 1 | 1 |
| 0 | 7 | 1 | 1 | 4 | 4 | 2 |
| 0.5 | 7 | 1 | 1 | 1 | 1 | 3 |
| 0.5 | 5.5 | 2 | 2 | 3 | 4 | 3 |
|  | 7 | 1 | 1 | 1 | 3 | 3 |
| 1.5 | 7 | 1 | 1 | 1 | 1 | 1 |
| 4 | 6 | 2 | 2 | 2 | 2 | 2 |
| 0 |  | 1 | 1 | 1 | 1 | 3 |
| 1 | 6.5 | 2 | 4 | 3 | 5 | 5 |
| 8 | 7 | 1 | 1 | 1 | 1 | 5 |
| 4 | 7 | 2 | 1 | 3 | 4 | 3 |
| 0 | 6 | 1 | 1 | 1 | 1 | 1 |
| 1 | 7 | 1 | 1 | 3 | 2 | 1 |
|  | 6 | 1 | 1 | 1 | 2 | 1 |
| 1 | 6 | 2 | 2 | 1 | 3 | 4 |
| 8 | 7 | 1 | 3 | 2 | 3 | 5 |
| 1.5 | 7.5 | 2 | 2 | 1 | 3 | 1 |
|  | 6 | 2 | 1 | 1 | 2 | 3 |
|  | 6 | 3 | 2 | 5 | 3 | 5 |
| 0 | 12 | 1 | 1 | 1 | 1 | 1 |
|  | 6 | 1 | 1 | 1 | 3 | 4 |
|  |  | 1 | 1 | 1 | 1 | 1 |
| 0.5 | 6.5 | 1 | 1 | 1 | 1 | 1 |
| 6 | 7 | 1 | 1 | 1 | 1 | 4 |
|  | 6.5 | 1 | 1 | 1 | 1 | 2 |
|  | 5 | 1 | 1 | 1 | 2 | 3 |
|  | 6 | 2 | 2 | 3 | 3 | 4 |
|  | 6 | 1 | 1 | 2 | 1 | 3 |
| 0 | 9 | 2 | 1 | 4 | 4 | 5 |
|  |  | 4 | 1 | 1 | 1 | 3 |
|  | 6.5 | 1 | 1 | 1 | 1 | 1 |
|  | 7 | 1 | 1 | 1 | 1 | 1 |
| 2 | 6 | 1 | 1 | 1 | 3 | 3 |
| 1 | 7 | 3 | 2 | 2 | 2 | 2 |
|  | 6 | 1 | 1 | 1 | 1 | 1 |
| 0 | 7 | 1 | 1 | 3 | 4 | 2 |
| 4 | 7.5 | 1 | 1 | 1 | 1 | 1 |
| 1.5 | 5.5 | 4 | 2 | 2 | 5 | 4 |
| 0.5 | 5.5 | 2 | 2 | 4 | 5 | 4 |
| 0.5 | 8 | 1 | 1 | 1 | 1 | 1 |
| 2 | 7.5 | 2 | 1 | 2 | 3 | 4 |
|  | 6.5 | 1 | 1 | 1 | 1 | 1 |
|  | 8 | 1 | 1 | 1 | 1 | 1 |
|  |  | 1 | 1 | 1 | 1 | 1 |
| 0.5 | 7 | 2 | 2 | 1 | 1 | 4 |
|  | 7.5 | 1 | 1 | 1 | 1 | 2 |
|  | 6 | 2 | 2 | 4 | 2 | 5 |
| 4 | 7 | 1 | 1 | 1 | 1 | 2 |
| 2 | 8 | 1 | 1 | 2 | 2 | 4 |
| 0 | 8 | 2 | 2 | 2 | 3 | 3 |
|  | 8 | 1 | 2 | 1 | 2 | 3 |
|  | 7 | 2 | 2 |  | 4 | 4 |
| 4 | 6 | 2 | 2 | 2 | 2 | 4 |
| 4 | 6 | 1 | 1 | 1 | 1 | 1 |
| 0 | 7 | 1 | 1 | 1 | 1 | 1 |
|  | 7 | 2 | 3 | 3 | 2 | 4 |
|  | 6 | 1 | 1 | 1 | 1 | 1 |
|  | 10 | 3 | 3 | 1 | 1 | 1 |
| 7 | 8 | 1 | 1 | 1 | 1 | 1 |
|  | 6.5 | 1 | 1 | 1 | 3 | 5 |
| 0 | 6 | 1 | 1 | 2 | 1 | 1 |
| 7 | 7 | 1 | 1 | 1 | 2 | 1 |
| 4 | 8 | 1 | 1 | 1 | 1 | 1 |
| 4 | 7 | 2 | 1 | 1 | 2 | 1 |
| 1.5 | 7 | 1 | 2 | 1 | 4 | 4 |
| 0.5 | 6 | 2 | 1 | 3 | 1 | 5 |
| 0 | 7 | 1 | 1 | 3 | 2 | 3 |
|  | 8 | 1 | 1 | 1 | 1 | 1 |
| 0 | 6 | 1 | 1 | 1 | 1 | 1 |
|  |  | 1 | 1 | 1 | 1 | 1 |
| 2 | 6.5 | 1 | 1 | 1 | 2 | 3 |
| 0 | 6 | 2 | 2 | 2 | 3 | 3 |
|  | 8 | 2 | 1 | 1 | 1 | 1 |
| 6 | 8 | 1 | 1 | 1 | 2 | 3 |
|  | 6 | 2 | 2 | 4 | 2 | 3 |
| 1.5 | 6 | 1 | 1 | 1 | 2 | 1 |
|  |  | 1 | 1 | 1 | 3 | 2 |
| 2 | 6.5 | 1 | 1 | 1 | 2 | 3 |
| 1 | 6 | 3 | 2 | 4 | 4 | 4 |
|  |  | 1 | 1 | 3 | 1 | 5 |
| 3 | 7.5 | 1 | 1 | 1 | 2 | 2 |
|  | 5 | 2 | 1 | 2 | 2 | 4 |
| 6 | 7 | 2 | 2 | 1 | 1 | 2 |
| 0 | 2.5 | 1 | 1 | 4 | 5 | 5 |
|  | 7 | 1 | 1 | 1 | 1 | 1 |
| 0 | 6 | 1 | 1 | 2 | 4 | 1 |
| 0 | 6.5 | 5 | 1 | 5 | 1 | 5 |
|  | 8 | 1 | 1 | 1 | 1 | 1 |
|  | 8 | 1 | 1 | 1 | 2 | 2 |
| 1 | 7 | 1 | 1 | 1 | 1 | 2 |
| 7.5 | 7.5 | 1 | 1 | 1 | 1 | 3 |
|  | 6 | 1 | 1 | 1 | 3 | 4 |
| 2.5 | 6.5 | 3 | 2 | 2 | 5 | 2 |
| 0 | 8 | 2 | 1 | 1 | 2 | 2 |
|  | 6 | 3 | 1 | 1 | 2 | 1 |
|  | 6 | 2 | 2 | 2 | 3 | 2 |
|  | 7 | 1 | 1 | 1 | 1 | 1 |
|  | 7.5 | 2 | 2 | 1 | 4 | 2 |
| 2 | 8 | 2 | 2 | 2 | 3 | 3 |
| 2 | 6 | 1 | 1 | 2 | 3 | 4 |
|  | 7 | 1 | 1 | 1 | 1 | 1 |
| 1.5 | 6 | 3 | 3 | 4 | 4 | 5 |
|  | 8 | 2 |  | 1 | 2 | 1 |
| 0.5 | 7.5 | 1 | 1 | 1 | 1 | 2 |
| 1 | 7 | 1 | 1 | 2 | 3 | 2 |
|  | 6 |  | 1 | 1 | 3 | 4 |
| 2 | 7 | 1 | 1 | 3 | 1 | 1 |
|  | 8.5 | 1 | 1 | 1 | 1 | 1 |
|  | 5 | 3 | 1 | 1 | 3 | 2 |
| 8 | 8 | 1 | 1 | 1 | 1 | 1 |
| 4 | 7 | 1 | 1 | 2 | 2 | 4 |
| 0 | 6.5 | 3 | 4 | 4 | 4 | 5 |
|  | 7.5 | 1 | 1 | 1 | 1 | 1 |
| 4 | 7 | 2 | 1 | 5 | 2 | 5 |
| 0 | 5 | 4 | 2 | 4 | 4 | 4 |
|  | 8 | 1 | 1 | 1 | 1 | 1 |
|  | 8 | 3 | 2 | 2 | 4 | 2 |
| 0 | 6 | 1 | 1 | 1 | 1 | 1 |
| 2 | 5.5 | 1 | 1 | 1 | 1 | 2 |
| 0 | 7 | 1 | 1 | 1 | 1 | 1 |
| 1 | 5.5 | 2 | 1 | 2 | 3 | 4 |
| 2 | 6 | 3 | 1 | 4 | 5 | 2 |
|  | 8 | 1 | 1 | 1 | 1 | 1 |
| 1.5 | 6.5 | 1 | 1 | 3 | 3 | 5 |
|  | 8 | 1 | 1 | 1 | 1 | 1 |
|  | 5 | 2 | 2 | 3 | 4 | 4 |
